# Botulinum Toxin Injection with Conjunctival Microincision for the Treatment of Acute Acquired Comitant Esotropia and Its Effectiveness

**DOI:** 10.1155/2020/1702695

**Published:** 2020-12-31

**Authors:** Hongjia Xu, Weifeng Sun, Shuying Dai, Yanyan Cheng, Jing Zhao, Yuan Liu, Juan Wang, Ya'nan Wang, Yu Gao, Huifang Han, Aijun Han

**Affiliations:** Department of Ophthalmology, Hebei Provincial Key Laboratory of Ophthalmology, Hebei Eye Hospital, Xingtai, Hebei Province 054000, China

## Abstract

**Purpose:**

To report on an improved botulinum toxin injection with conjunctival microincision for beginners, and to determine the effectiveness of botulinum toxin A (BTXA) in the treatment of patients with acute acquired comitant esotropia (AACE).

**Methods:**

Medical records of 29 AACE patients were retrospectively analyzed. BTXA was injected into the unilateral or bilateral medial rectus muscle with conjunctival microincision without electromyographic guidance. Success was defined as total horizontal deviation ≤10 prism diopters (PD) and evidence of binocular vision.

**Results:**

Twenty-nine patients were included, of whom 22 were male and 7 were female. The mean age at onset was 14.2 ± 7.4 (range, 4–34) years. The mean time from onset of AACE to injection was 18.4 ± 20.3 (range, 1–96) weeks. All patients completed at least 6 months of follow-up, and the mean follow-up after BTXA injection was 12.3 ± 4.8 months (range, 7–24 months). Neurological evaluation and brain magnetic resonance imaging (MRI) were unremarkable in all patients. The mean spherical equivalent refraction was −1.22 ± 2.85D and −0.97 ± 2.80D in the right and left eyes, respectively. Mean preinjective esotropia was 38.4 ± 18.9 PD (range, +10–+80 PD) at near and 40.2 ± 17.7 PD (range, +20–+80 PD) at far distance. The mean angle of deviation at 6 months after injection was 0.6 ± 4.1 PD (range, −3–+15 PD) at near and 3.0 ± 5.9 PD (range, 0–+20 PD) at far distance. There was significant difference in the angle of deviation at near and far fixation between pre-BTXA and post-BTXA 6 months (*p* < 0.001, *p* < 0.001, resp.). There was no significant difference in the angle of deviation at near and far fixation between post-BTXA 6 months and post-BTXA at final follow-up (*p* = 0.259 and 0.326, resp.). Mean stereoacuity improved from 338 to 88 arc seconds. During the follow-up period, 5 of 29 patients had recurrent esotropia. Two patients refused all further treatment, and the other 3 patients required incisional strabismus surgery. The success rates were 86.2% (25/29) at 6 months and 82.8% (24/29) at final follow-up.

**Conclusion:**

Conjunctival microincision injection of botulinum toxin is a practical and safe method for beginners to locate an extraocular muscle, which is as effective as the traditional methods. Botulinum toxin injection can be preferred as the first-line treatment for AACE patients with potential binocular vision.

## 1. Introduction

Acute acquired comitant esotropia (AACE) is characterized by the sudden onset of a comitant esotropia with no evidence of lateral rectus paralysis [1]. For the treatment of AACE, there are currently prisms, strabismus surgery [1–4], and botulinum toxin type A (BTXA) injections [5, 6]. Strabismus surgery requires general anesthesia or sedation in most cases, whereas botulinum injection may be performed under topical anesthesia in older children and adults. In addition, strabismus surgery is more invasive than BTXA treatment and can cause muscle and conjunctival scarring. An increasing number of clinicians currently begin offering BTXA as first-line therapy for AACE [5].

For the injection of BTXA, there are electromyographic (EMG) guidance without conjunctival incision [6–8], injection under direct visualization without EMG guidance at the time of strabismus surgery [9–11], transconjunctival injection without conjunctival incision and EMG guidance [5, 12], etc. EMG guidance is more time-consuming and more expensive [13]. Botulinum toxin injection under direct vision is generally used as an assistant in strabismus surgery [10, 11], which can also be used in novice injectors as an independent treatment modality. But the method also causes conjunctival scarring. Injection without conjunctival incision and EMG guidance is difficult for novice injectors, which may cause potential complications such as ocular perforation [14–16].

The purpose of this study was to report on the botulinum toxin injection with conjunctival microincision, a safer method for beginners to inject BTXA into the extraocular muscle (EOM) without EMG guidance, and to investigate the efficacy of BTXA in the treatment of patients with AACE.

## 2. Materials and Methods

We retrospectively reviewed records of 29 patients diagnosed with AACE from April 2017 to January 2019 at Hebei Eye Hospital in North China. Our study was conducted in adherence to the tenets of the Declaration of Helsinki and approved by the ethics committee of the hospital. Informed consent was obtained from the participants and their caregivers (legal guardian).

Patients who met the following criteria were included in this study: (1) age of onset >3 years; (2) acute onset of comitant strabismus (deviation difference was less than 5 prism diopters (PD) in any field of gaze at distance); (3) normal eye movement; (4) hyperopia < +2.50 DS (spherical equivalent); (5) at least 6 months of follow-up after treatment. Patients were excluded if there was a known history of strabismus, evidence of neurologic abnormality, or acute incomitant esotropia.

A comprehensive medical history was taken, including general health, birth history, family history, and previous ocular history. The history of squinting and symptoms related to onset was recorded. All patients underwent ophthalmological, orthoptic, and neurologic examinations and had brain magnetic resonance imaging (MRI).

Distant Vision was examined through E Standard Logarithm Eyesight Table. Cycloplegic refraction was performed after administering 1% atropineointment three times a day for 3 days in patients younger than 7 years and 1% cyclopentolate eye drops every 5 min for three times for those ≥7 years old. Spherical equivalents (SE) of refractive error were calculated by using the algebraic sum of the dioptric powers of the sphere and half of the cylinder. Ocular motility was evaluated clinically. The angle of deviation at near (1/3 m) and far (6 m) distance fixation was measured using the prism and alternate cover tests with and without refractive correction. Near stereoacuity was measured at 40 cm using the Titmus fly test with best refractive correction but without prism correction.

Botulinum toxin was first introduced to our group for this indication in 2015. All injections were performed by one injector (WFS) without EMG guidance. The injection was performed under general anesthesia in patients under 12 years old, and the remainder, who were able to tolerate injection under topical anesthesia. Topical proparacaine hydrochloride and epinephrine 0.01% were used in all patients before injection. The epinephrine reduces the likelihood of conjunctival haemorrhage and allows the anterior ciliary vessels to be seen more clearly, providing a useful guide to the location of the medial rectus. Botulinum toxin A for injection (BTXA, 50 units lyophilisate vials, Lanzhou Institute of Biological products, Lanzhou, China) was dissolved in 2 ml of normal saline before injection, so a volume of 0.1 ml liquid contained 2.5 units of BTXA.

We chose to inject botulinum toxin into the unilateral medial rectus muscle of the deviating eye when the mean angle was 15 PD to 20 PD [6] and bilateral medial rectus muscle when the mean angle was 20 PD to 80 PD. The dose of botulinum toxin was 2.5 units into the unilateral medial rectus muscle of the deviating eye [6]. The dose of botulinum toxin was 5 units into both medial rectus muscles (2.5 units per muscle) when the angle of deviation was 20 PD to 40 PD, 6 units (3 units per muscle) when the angle of deviation was 40 PD to 60 PD, and 7 units (3.5 units per muscle) when the angle of deviation was 60 PD to 80 PD (Table 1).

**Figure 1:**
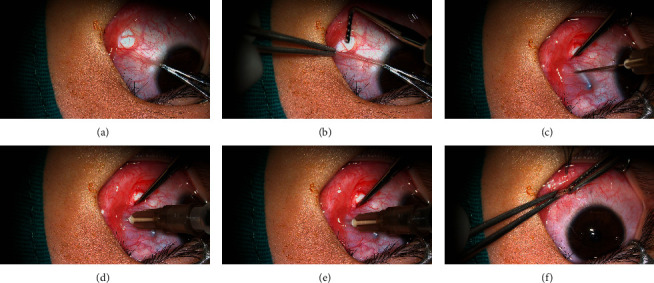
Procedures of conjunctival microincision injection of botulinum toxin.

We disinfected the periorbital skin with 5% povidone iodine solution before injection. After eyelid speculum insertion, disinfect the conjunctival sac with 0.05% povidone iodine solution for 1 minute. The procedure was performed by pulling the eye into an abducted position. About 2 mm microincision was made close to the plica semilunaris at inferior nasal quadrants through conjunctiva and Tenon's capsule. Medial rectus was held on a strabismus hook, and a 1 mL syringe with a 15 mm, 26-gauge needle, with the bevel of the needle toward the sclera, was introduced into the conjunctiva 6 mm from the limbus into the belly of the medial rectus muscle and advanced approximately 15 mm along the muscle path. The syringe was moved up and down parallel to the attachment point of medial rectus muscle while the needle advanced to make sure the needle tip inside the muscle and to prevent it from perforating the sclera. The conjunctival microincision was closed with Electrocoagulation hemostat. Finally, rinse conjunctival sac with normal saline. The procedure of conjunctival microincision injection of botulinum toxin is shown in Figure 1.

Success was defined as (i) a final horizontal deviation of 10 PD or less by alternate prism cover testing at distance, (ii) with evidence of binocular vision and (iii) no need for retreatment (e.g., any further procedures, strabismus surgery, or botulinum toxin injection for a horizontal deviation) [17]. Binocular vision was defined as gross stereopsis on the Titmus fly test in our study.

SPSS version 17.0 (SPSS Institute Inc., Chicago, IL, USA) was used for statistical analyses. A *t*-test was used to compare the angle of deviation at near and distance fixations. The paired *t*-test was used to compare the angle of deviation before and after BTXA injection. A *p* value of <0.05 was considered statistically significant.

## 3. Results

Twenty-nine patients met the inclusion criteria of AACE, and within the group there were twenty-two males and seven females.  Table 2 summarizes the data for each patient. The mean age at onset of AACE was 14.2 ± 7.4 years (range, 4–34 years). The duration from onset of the deviation to time of injection was 18.4 ± 20.3 weeks (range, 1–96 weeks). All patients completed at least 6 months of follow-up, and the mean follow-up after BTXA injection was 12.3 ± 4.8 months (range, 7–24 months) (Table 3). Results of neurological examination as well as brain MRI were normal.

Among the 29 patients, one patient was emmetropic, 14 patients were myopic (spherical equivalent, range: −0.50 DS to −7.50 DS), and 14 patients were mild hyperopic (spherical equivalent, range: +0.50 DS to +2.25 DS). The mean spherical equivalent refraction was −1.22 ± 2.85 D and −0.97 ± 2.80 D in the right and left eyes, respectively (Table 3). The best corrected visual acuity was 20/20 or better in both eyes of patients ≥6 years old, and 20/25 or better for the patients <6 years old. Children with hypermetropia ranging from a spherical equivalent of +1.50 DS to +2.25 DS were prescribed the full spectacle correction full time for a minimum of 4 weeks before BTXA injection. Among these children, there was no significant difference in the angle of esodeviation for near or far distance before and after wearing the glasses.

The pre-BTXA angle of deviation was 38.4 ± 18.9 PD (range, +10–+80 PD) at near and 40.2 ± 17.7 PD (range, +20–+80 PD) at far distance with full correction of refractive errors. There was no significant difference between far and near fixation (*p*=0.202). The Titmus fly test showed that there were 13 patients with normal near stereoacuity (≤63″) and 16 patients with abnormal near stereoacuity. Mean preinjective stereoacuity was 338 ± 749 (range, 20–3000) arc seconds. The angle of deviation at 6 months after injection was 0.6 ± 4.1 PD (range, -3–+15 PD) at near and 3.0 ± 5.9 PD (range, 0–+20 PD) at far distance. There was significant difference in the angle of deviation at near and far fixation between pre-BTXA and post-BTXA 6 months (*p* < 0.001, *p* < 0.001, resp.). Mean esodeviation at final follow-up after injection was 1.7 ± 6.8 PD (range, −3–+30 PD) at near and 3.9 ± 7.1 PD (range, 0–+25 PD) at far distance. There was no significant difference in the angle of deviation at near and far fixation between post-BTXA 6 months and post-BTXA at final follow-up (*p* = 0.259 and 0.326, resp.). Pre- and post-BTXA comparison was described in Tables 4 and 5.

Patients with small angle esotropia (15∼20 PD) were not overcorrected during the early postoperative period and maintained excellent ocular alignment and high-grade stereopsis without further intervention. During the follow-up period, 5 of 29 patients failed treatment: 4 patients had recurrent esotropia at 6 months, and 1 patient (patient 13 on the summary chart) considered treatment success at 6 months developed recurrent esotropia at 10 months. Patient 29 and the parents of patient 1 refused further intervention with toxin or surgery, and the other 3 patients (patients 13, 24, and 28 on the summary chart) required incisional strabismus surgery: patient 13 with a 60 PD deviation was treated with unilateral medial rectus recession and lateral rectus resection 16 months after the BTXA injection; both patient 24 and patient 28 with a 25 PD deviation underwent unilateral medial rectus recession of the deviating eye 13 months after the BTXA injection. However, the five patients maintained the same stereopsis at the last follow-up as before the injection. Among the 24 patients who did not have recurrent esotropia at final follow-up, 15 had improved stereopsis and 14 had high-grade stereoacuity of 63 seconds of arc or better. Compared with before injection, no patient's stereopsis was further damaged. Mean stereoacuity improved from 338 to 88 arc seconds. The success rates were 86.2% (25/29) at 6 months and 82.8% (24/29) at final follow-up.

**Figure 2:**
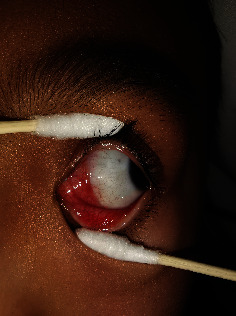
The healing of the conjunctival microincision.

There were no serious or permanent complications. The common complications in this study were transient postoperative exotropia (*n* = 7, 24%), hypertropia (*n* = 4, 14%), and ptosis (*n* = 3, 10%), which resolved within 1 month. There were seven patients with transient overcorrection, with 20–60 prism diopters of exotropia as a consequence of botulinum injection. There were no serious complications, such as scleral perforation, vitreous haemorrhage, and retinal detachment. There was no visible conjunctival scar after healing (Figure 2).

## 4. Discussion

Acute acquired comitant esotropia (AACE) is characterized by the acute onset of comitant esotropia that often occurs after binocular vision well developed in older children and adults. The primary treatments for AACE include conventional horizontal rectus muscle surgery [1–4] and BTXA injection [5, 6].

BTXA has been used for the treatment of strabismus for nearly 40 years [18]. BTXA, an injectable neurotoxin, selectively acts on peripheral cholinergic nerve terminals at the neuromuscular junction to block acetylcholine release, inhibiting muscle contraction [19].This neurotoxin induces temporary severe muscle paralysis without damaging the rectus muscle or the peripheral nerve [7, 11]. Although the toxin loses activity in approximately 3 months, it has been demonstrated that there can be a long-lasting effect on ocular alignment [20]. This is likely because temporary paralysis results in changes in the length and tension of injected and antagonist muscles. Another possible explanation is that botulinum toxin gives the binocular visual system the opportunity to reestablish a normal fusion mechanism for patients with binocular vision, which can be maintained when its effect disappears [7].

Compared with strabismus surgery, botulinum toxin therapy has several potential advantages. There is little damage to extraocular muscles, anterior ciliary circulation, and conjunctiva, which makes future surgeries easier. BTXA treatment can shorten the time of general anesthesia in children, and there is no pain after the operation. Botulinum toxin may help to minimize the time from onset to treatment. In our hospital, we recommend that AACE patients with binocular vision should give priority to BTXA injection treatment, and for patients who refuse BTXA injection treatment, surgical treatment should be performed only when the angle of deviation is stable. There are few studies on the long-term impact of human anomalous binocular vision experience on stereopsis. Fawcett et al. suggest that the critical period for susceptibility of human stereopsis to an anomalous binocular visual experience is at least before the age of 4.6 years [21]. Temporary exotropia may occur after BTXA injection for different periods of time. It is not clear if the total time of anomalous binocular experience is different (time between onset and injection plus duration of postinjection exotropia, versus time between onset and strabismus surgery).

Botulinum toxin is delivered by injection to the site of disease. There are currently three methods of BTXA injection into the extraocular muscle (EOM): EMG guidance [6–8], direct vision injection without EMG guidance [9–11], and injection without conjunctival incision and EMG guidance [5, 12].

Scott recommends EMG guided injection [22], which is inserting the electrode needle into the EOM, and injecting when characteristic cracking noise was heard. Injection using EMG guidance is to help locate the EOM and nerve motor endplate to ensure that the toxin is accurately delivered to the target muscle [23, 24]. Suppressed muscle activity under general anesthesia prevents measurement of EMG of the EOM. The EMG guidance of botulinum toxin injection has some disadvantages limiting its wide use, such as the high cost of the appliance, the use of disposable special needles in addition to the need to use the ketamine anesthetic when applying in children [25].

Botulinum toxin injection under direct visualization of the muscle and without EMG guidance is generally used as an assistant in strabismus surgery to improve outcomes in patients with large-angle esotropia [10, 11]. This method can be used in novice injectors as an independent treatment modality. But the procedure can also cause conjunctival scarring.

Injection without conjunctival incision and EMG guidance is administered by pulling the eye into an abducted position and then positioning the tip of the needle at the temporal border of the plica semilunaris, piercing the conjunctiva, and advancing the needle approximately 2 cm along the muscle path. Alternatively, the muscle may be grasped transconjunctivally with forceps before injecting [25]. The need to inject near the ocular surface of the muscle (to block the nerves which enter from the bulbar surface) takes one closer to the sclera which is already thin below the muscle belly [26]. Risks during injections include scleral perforation, vitreous haemorrhage, and retinal detachment [14–16].

Sanjari et al. reported that the efficacy of botulinum toxin injection into the medial rectus muscle for treatment of abducens nerve palsy was the same when EMG was used or not [12]. Benabent et al. found that the effect of botulinum toxin injection in the treatment of congenital esotropia without EMG was not significantly different from that of other EMG guided studies [27].

We use conjunctival microincision to apply BTXA. The microincision was close to the plica semilunar, and there was no visible scar in the conjunctiva after healing (Figure 2). During the injection, hold the strabismus hook in one hand and the syringe in the other. By moving the syringe, we can feel whether the tip of the needle is advancing in the muscle, which, in turn, may theoretically reduce the risk of ocular perforation.

There are very few studies on the use of botulinum toxin for AACE. Our results are compatible with published observation studies of the use of botulinum toxin for the treatment of AACE [5, 6]. Dawson and associates looked at 14 patients treated with botulinum toxin under EMG control. They reported that the success rate was 79% after a mean follow-up period of 22 months, and the incidences of ptosis and exotropia were 36% and 71%, respectively [6].Wan, Mantagos, and associates reported success rates of 81% at 6 months and 67% at 18 months using an injection of botulinum toxin without conjunctival incision and EMG guidance in the treatment of patients with AACE. The incidences of ptosis and exotropia were 50% and 56%, respectively [5].

According to the traditional view, small to moderate angle strabismus particularly benefits from the use of botulinum toxin, and large-angle esotropia (>60 PD) is generally treated by strabismus surgery or BTXA-augmented surgery. In our study, there were 4 patients with large-angle esotropia whose parents refused strabismus surgery and strongly requested botulinum toxin injection. The dose of botulinum toxin was 7 units into bilateral medial rectus in these 4 patients. Surprisingly, up to the last follow-up, the orthophoric position and improved stereopsis were achieved in the 4 patients after toxin therapy.

Our study has some limitations, including its retrospective design and small sample size. Additionally, our subjects were followed up for a minimum period of 7 months, and therefore further studies with longer-term follow-up should be performed to further determine the long-term stability of eye alignment after BTXA treatment. Further, the cause of recurrence of AACE in 5 cases is still unclear. No obvious abnormality was found in brain MRI, and no nystagmus and papilledema were found. Cai et al. found that the distance from the insertion of medial rectus to limbus was shorter in patients with AACE [28]. Reasons for the recurrence of AACE after botulinum toxin treatment remain an open question, and longer-term studies are needed to provide more definitive answers. Finally, since our method involves pulling the medial rectus muscle to the temporal side with a strabismus hook, the subjective information about the comfort and fear degree of patients and the objective information about the oculocardiac reflex should also be recorded.

## 5. Conclusions

In conclusion, botulinum toxin treatment in our series had a good success rate. We suggest that conjunctival microincision injection of botulinum toxin is a practical and safe method for beginners to locate an EOM, which is as effective as the traditional methods. Botulinum toxin injection can be a first-line treatment for AACE patients with potential binocular vision.

## Figures and Tables

**Table 1:** Botulinum toxin dosage.

Esotropia (PD)	Dose of botulinum toxin (units)
15∼20	2.5
20∼40	5
40∼60	6
60∼80	7

Data are the total dosage of botulinum toxin per visit. PD: prism diopters.

**Table 2:** Summary of patient data.

Patient no.	Sex	Age at Onset（years）	Onset to BTXA (wks)	BCVA,Rt/Lt	Cycloplegic refraction(SE)		Angle pre-BTXA (PD)		Angle 6 mos post-BTXA (PD)		Final angle (PD)		Stereo pre-BTXA	Stereo at 6 mos	Final stereo	Follow-up period (mos)	Recurrence
					Rt(D)	Lt(D)	Near	Distance	Near	Distance	Near	Distance					
1	M	4	5	0.8/0.8	+1.75	+2.25	30	30	0	15	0	15	40	40	40	17	+
2	M	4	8	0.8/0.8	+1.50	+1.50	60	50	−2	0	−2	0	100	32	32	24	−
3	M	6	20	0.8/0.8	+2.00	+2.00	50	60	0	0	0	0	3000	100	100	18	−
4	M	6	3	1.0/1.0	+1.25	+1.25	50	50	0	0	0	0	100	25	25	11	−
5	M	7	5	0.8/0.8	+2.00	+2.00	40	40	0	3	0	3	400	100	100	8	−
6	F	8	5	1.0/1.0	+2.00	+2.00	70	80	0	0	0	0	400	100	100	12	−
7	M	8	12	1.0/1.0	+1.75	+2.25	40	50	0	0	0	0	200	100	100	10	−
8	M	8	4	1.0/1.0	+1.00	+1.00	50	60	0	0	0	0	63	32	32	18	−
9	M	9	24	1.0/1.0	+0.75	+0.75	80	60	0	0	0	0	400	100	100	7	−
10	M	10	1	1.0/1.0	+0.50	+0.50	40	40	0	0	0	0	100	32	32	12	−
11	M	12	8	1.0/1.0	+1.25	+1.50	20	25	−3	0	−3	0	160	160	160	8	−
12	F	12	8	1.0/1.0	−0.50	+0.50	20	25	−2	0	0	0	32	25	25	8	−
13	M	12	12	1.0/1.0	−4.00	−3.75	40	30	0	0	30	25	160	160	160	10	+
14	M	13	12	1.0/1.0	−2.75	−1.00	50	50	0	9	0	9	160	160	160	7	−
15	M	13	48	1.0/1.0	−4.50	−4.25	40	40	0	0	0	0	160	40	40	7	−
16	M	16	24	1.0/1.0	−3.75	−3.25	25	30	−2	0	−2	0	50	50	50	17	−
17	M	16	12	1.0/1.0	−2.75	0.00	40	30	0	0	0	0	400	50	50	13	−
18	M	16	1	1.0/1.0	0.00	0.00	30	30	0	0	0	0	400	400	400	18	−
19	M	17	12	1.0/1.0	−7.50	−7.50	30	30	−2	0	−2	0	50	50	50	12	−
20	M	17	1	1.0/1.0	+1.00	+1.25	75	80	0	0	0	0	3000	400	400	16	−
21	F	17	16	1.0/1.0	−3.00	−3.75	10	20	0	0	0	0	50	32	32	9	−
22	M	17	48	1.0/1.0	−4.75	−4.50	15	20	3	8	3	8	25	25	25	9	−
23	M	17	96	1.0/1.0	+1.50	+1.00	50	50	0	0	0	0	32	32	32	9	−
24	F	18	24	1.0/1.0	−3.75	−3.50	60	65	15	15	15	15	25	25	25	7	+
25	M	18	4	1.0/1.0	−3.75	−3.25	20	20	0	3	0	3	32	25	25	17	−
26	M	19	24	1.0/1.0	−3.50	−3.25	15	25	−2	0	−2	0	63	63	63	9	−
27	F	29	48	1.0/1.0	−1.50	−1.50	15	20	−2	0	−2	0	160	160	160	19	−
28	F	30	28	1.0/1.0	−1.00	−2.25	30	30	15	20	15	20	20	20	20	7	+
29	F	34	20	1.0/1.0	−6.75	−6.25	20	25	0	15	0	15	20	20	20	19	+

F: female, M: male, BTXA: botulinum toxin A, BCVA: best corrected visual acuity, Rt: right, Lt: left, SE: spherical equivalent, D: diopter, PD: prism diopters.

**Table 3:** Clinical profile of study population.

Variable	Mean	Standard deviation	Range
Age at onset (years)	14.2	7.4	4–34
Onset to BTXA(wks)	18.4	20.3	1–96
Spherical equivalent right eye	−1.22	2.85	−7.50 to +2.00
Spherical equivalent left eye	−0.97	2.80	−7.50 to +2.25
Follow-up period (mos)	12.3	4.8	7–24

BTXA: botulinum toxin A.

**Table 4:** Pre- and Post-BTXA 6 months comparison in patients with AACE.

		Pre-BTXA	Post-BTXA 6 months	*p*
Angle of deviation (PD)	Near	38.4 ± 18.9	0.62 ± 4.1	<0.001
	Distance	40.2 ± 17.7	3.0 ± 5.9	<0.001

BTXA: botulinum toxin A, PD: prism diopters.

**Table 5:** Post-BTXA 6 months and Post-BTXA at final follow-up comparison in patients with AACE.

		Post-BTXA 6 months	Post-BTXA at final follow-up	*p*
Angle of deviation (PD)	Near	0.62 ± 4.1	1.7 ± 6.8	0.259
	Distance	3.0 ± 5.9	3.9 ± 7.1	0.326

BTXA: botulinum toxin A, PD: prism diopters.

## Data Availability

The data used to support the findings of this study are restricted by the ethics committee of Hebei Eye Hospital in order to protect patient privacy.
